# Supplementary material for: A curative combination cancer therapy achieves high fractional cell killing through low cross-resistance and drug additivity
Source: eLife. 2019 Nov 19;8:e50036. doi: 10.7554/eLife.50036 (PMC6897534; doi:10.7554/eLife.50036)
Supplement: Transparent reporting form [file elife-50036-transrepform.pdf]

eLIFE
1st Floor
24 Hills Road
Cambridge CB2 1JP, UK
P 01223 855340
W elifesciences.org
T @elife

eLife's transparent reporting form

We encourage authors to provide detailed information *within their submission* to facilitate the interpretation and replication of experiments. Authors can upload supporting documentation to indicate the use of appropriate reporting guidelines for health-related research (see EQUATOR Network), life science research (see the BioSharing Information Resource), or the ARRIVE guidelines for reporting work involving animal research. Where applicable, authors should refer to any relevant reporting standards documents in this form.

If you have any questions, please consult our Journal Policies and/or contact us: editorial@elifesciences.org.

Sample-size estimation

- You should state whether an appropriate sample size was computed when the study was being designed
- You should state the statistical method of sample size computation and any required assumptions
- If no explicit power analysis was used, you should describe how you decided what sample (replicate) size (number) to use

Please outline where this information can be found within the submission (e.g., sections or figure legends), or explain why this information doesn't apply to your submission:

Dose-response measurements did not involve a power analysis; we observed that quadruplicate measurements with ~1.5-fold increments in drug concentration provided sufficiently narrow 95% confidence intervals in potency to identify deviations from additivity of magnitude 2-fold or greater, which is an editorially defined threshold for meaningful synergy (FC Odds, 2003, J Antimicrob Chemother). Clone tracing experiments were preceded by simulations studies (described in Methods) which determined that >99.99% coverage of 10^6 barcodes would be achieved with populations of >12*10^6 cells per flask. The choice of 10^6 barcodes was determined by practical limits on the volumes of cultures that could be propagated with >20 independent cultures. The choice of biological triplicates per drug treatment was informed by a prior clone-tracing study (HE Bhang et al, 2015, Nature Medicine) which found a majority of drug-resistant clones to be reproducible in a majority of replicates. CRISPR screens were performed maintaining a minimum coverage of 300 cells per sgRNA at all steps; a minimum of 100 is recommended to avoid stochastic noise (Nagy & Kampmann, 2017, BMC Bioinformatics). Internal replicates in the form of 10 sgRNAs per gene target is the best presently available genome-wide sgRNA libraries.

Replicates

- You should report how often each experiment was performed
- You should include a definition of biological versus technical replication
- The data obtained should be provided and sufficient information should be provided to indicate the number of independent biological and/or technical replicates
- If you encountered any outliers, you should describe how these were handled
- Criteria for exclusion/inclusion of data should be clearly stated
- High-throughput sequence data should be uploaded before submission, with a private link for reviewers provided (these are available from both GEO and ArrayExpress)

Please outline where this information can be found within the submission (e.g., sections or figure legends), or explain why this information doesn't apply to your submission:

(i) Dose-response measurements consisted of repeat experiments performed on different days, each with biological repeats conducted on two independently propagated cultures, and technical repeats per culture of all single-drug dose responses. Thus, n=8 for monotherapy responses (2 x 2 biological repeats x 2 technical repeats) and n=4 for combination therapy responses (2 x 2 biological repeats). Outliers: in some experiments automated microplate dispensing systems failed to seed many wells of a microtiter plate, in which case the entire plate was discarded and the experiment repeated. All analysis was performed by software and involved no removal of outliers (beyond the aforementioned dismissal of an entire plate). See methods and figure captions. (ii) Clone tracing experiments consisted of triplicate independently propagated cultures, thus, n=3 biological repeats. All analysis was performed by software and involved no removal of outliers. See main text, methods and figure captions. (iii) CRISPR screens consisted of internal 10-fold replicates in the form of 10 different sgRNAs per gene target. All analysis was performed by software and involved no removal of outliers. See main text, methods, and figure captions.

eLife Sciences Publications, Ltd is a limited liability non-profit non-stock corporation incorporated in the State of Delaware, USA, with company number 5030732, and is registered in the UK with company number FC030576 and branch number BR015634 at the address 1st Floor, 24 Hills Road, Cambridge CB2 1JP | August 2014

Statistical reporting

- Statistical analysis methods should be described and justified
- Raw data should be presented in figures whenever informative to do so (typically when N per group is less than 10)
- For each experiment, you should identify the statistical tests used, exact values of N, definitions of center, methods of multiple test correction, and dispersion and precision measures (e.g., mean, median, SD, SEM, confidence intervals; and, for the major substantive results, a measure of effect size (e.g., Pearson's r, Cohen's d)
- Report exact p-values wherever possible alongside the summary statistics and 95% confidence intervals. These should be reported for all key questions and not only when the p-value is less than 0.05.

Please outline where this information can be found within the submission (e.g., sections or figure legends), or explain why this information doesn't apply to your submission:

All error bars in the article are 95% confidence intervals. Statistical tests are described where applicable in the main text and figure captions. All reports of correlations describe both effect size and significance. Methods sections describe the basis for calculating False Discovery Rates (FDR) in clone-tracing experiments and CRISPR screens.

(For large datasets, or papers with a very large number of statistical tests, you may upload a single table file with tests, Ns, etc., with reference to sections in the manuscript.)

Group allocation

- Indicate how samples were allocated into experimental groups (in the case of clinical studies, please specify allocation to treatment method); if randomization was used, please also state if restricted randomization was applied
- Indicate if masking was used during group allocation, data collection and/or data analysis

Please outline where this information can be found within the submission (e.g., sections or figure legends), or explain why this information doesn't apply to your submission:

Not applicable

Additional data files ("source data")

- We encourage you to upload relevant additional data files, such as numerical data that are represented as a graph in a figure, or as a summary table
- Where provided, these should be in the most useful format, and they can be uploaded as "Source data" files linked to a main figure or table
- Include model definition files including the full list of parameters used
- Include code used for data analysis (e.g., R, MatLab)
- Avoid stating that data files are "available upon request"

Please indicate the figures or tables for which source data files have been provided:

Source data for clone-tracing and CRISPR screens are available in Figure 4-source data 1 and Figure 5-source data 1,2,3 . Software for analysis of clone-tracing and CRISPR experiments was obtained from public sources described in Methods. Data used to construct graphs in Figure 6 and Figure 6-figure supplement 1 are available as Figure 6-source data 1.
